# Defining the roles of arrestin2 and arrestin3 in vasoconstrictor receptor desensitization in hypertension

**DOI:** 10.1152/ajpcell.00079.2015

**Published:** 2015-05-13

**Authors:** Jonathon M. Willets, Craig A. Nash, Richard D. Rainbow, Carl P. Nelson, R. A. John Challiss

**Affiliations:** ^1^Department of Cell Physiology and Pharmacology, University of Leicester, Leicester, United Kingdom; and; ^2^Department of Cardiovascular Sciences, University of Leicester, Glenfield General Hospital, Leicester, United Kingdom

**Keywords:** hypertension, G protein-coupled receptor, arrestin, vasoconstrictor, resistance artery, phospholipase C

## Abstract

Prolonged vasoconstrictor-stimulated phospholipase C activity can induce arterial constriction, hypertension, and smooth muscle hypertrophy/hyperplasia. Arrestin proteins are recruited by agonist-occupied G protein-coupled receptors to terminate signaling and counteract changes in vascular tone. Here we determine whether the development of hypertension affects arrestin expression in resistance arteries and how such changes alter arterial contractile signaling and function. Arrestin2/3 expression was increased in mesenteric arteries of 12-wk-old spontaneously hypertensive rats (SHR) compared with normotensive Wistar-Kyoto (WKY) controls, while no differences in arrestin expression were observed between 6-wk-old SHR and WKY animals. In mesenteric artery myography experiments, high extracellular K^+^-stimulated contractions were increased in both 6- and 12-wk-old SHR animals. Concentration-response experiments for uridine 5′-triphosphate (UTP) acting through P2Y receptors displayed a leftward shift in 12-wk, but not 6-wk-old animals. Desensitization of UTP-stimulated vessel contractions was increased in 12-wk-old (but not 6-wk-old) SHR animals. Dual IP_3_/Ca^2+^ imaging in mesenteric arterial cells showed that desensitization of UTP and endothelin-1 (ET1) responses was enhanced in 12-wk-old (but not 6-wk-old) SHR compared with WKY rats. siRNA-mediated depletion of arrestin2 for UTP and arrestin3 for ET1, reversed the desensitization of PLC signaling. In conclusion, arrestin2 and 3 expression is elevated in resistance arteries during the emergence of the early hypertensive phenotype, which underlies an enhanced ability to desensitize vasoconstrictor signaling and vessel contraction. Such regulatory changes may act to compensate for increased vasoconstrictor-induced vessel contraction.

hypertension is an important risk factor in the development of major cardiac, cerebral, and renal diseases. Hypercontractility of blood vessels can contribute to the hypertensive phenotype and present pharmacological approaches to combating hypertension focus, directly or indirectly, on the G protein-coupled receptors (GPCRs) within vascular smooth muscle regulating blood vessel diameter. Activation of the phospholipase C (PLC) pathway in arterial smooth muscle by a variety of contractile agonists (e.g., endothelin, angiotensin II, norepinephrine, extracellular nucleotides) increases intracellular Ca^2+^, which is of cardinal importance to their vasoconstrictor activity. Continuous or repeated agonist stimulation of Gα_q/11_-coupled GPCR signaling can have adverse effects on arterial smooth muscle cells (2, 12), suggesting that understanding the regulation of arterial smooth muscle Gα_q/11_ signaling might facilitate the identification of novel targets for the treatment of hypertension.

Continual or repeated agonist stimulation of GPCRs usually leads to reduced responsiveness to further agonist stimulation (25, 34), through the process of receptor desensitization, which has been shown to protect cells from the adverse effects of overstimulation and inappropriate GPCR signal transduction. Phosphorylation at key serine/threonine residues within the third intracellular loop and/or COOH-terminal tail of GPCRs is thought to be the primary event initiating receptor desensitization, and is mediated by a family of serine/threonine kinases, the G protein-coupled receptor kinases (GRKs) (34), of which up to five isoenzymes are expressed in vascular smooth muscle cells (3, 22). Phosphorylation by GRKs enhances receptor affinity for nonvisual arrestin2 and arrestin3 (also known as β-arrestin-1 and -2, respectively), which sterically hinder further interactions between receptor and G protein to inhibit G protein-dependent signal transduction.

GRK and arrestin proteins are important negative regulators of contractile signaling, and it might be expected that their expression increases in hypertension to counteract vasoconstrictor-stimulated vessel contraction. This is indeed the case; however, despite the fact that arterial smooth muscle cells express multiple isoenzymes of the GRK family (22), only GRK2 expression has been reported to be elevated in both hypertensive patients (8) and in rodent models of hypertension (9, 10). The selective upregulation of GRK2 is perhaps unsurprising considering the accumulating evidence that demonstrates a preeminent role for this GRK isoenzyme in regulating the function of a plethora of GPCRs activated by such diverse vasoconstrictors as endothelin-1 (ET1; via the ET_A_ receptor) (22), uridine 5′-triphosphate (UTP; via the P2Y_2_ receptor) (21), angiotensin II (ATII, via the AT_1_ receptor) (14), and norepinephrine (via α_1_-adrenergic receptors) (4) in arterial smooth muscle cells. Although mounting evidence suggests that vascular GRK2 upregulation may occur as an adaptive change to combat hypertension, an equivalent role for arrestin proteins has not been extensively investigated despite the fact that arrestins are reported to regulate the contractile signaling of several vasoconstrictors (14, 21, 22). To assess whether development of hypertension affects arrestin expression, we compared the expression of arrestin proteins in spontaneously hypertensive rats (SHR) at ages before (6 wk old) and after (12 wk old) hypertension has become established. We have, for the first time, demonstrated altered arrestin isoform expression in the resistance arteries of SHRs that is responsible for driving the enhanced desensitization of vasoconstrictor-mediated PLC signaling observed in hypertension.

## MATERIALS AND METHODS

### 

#### Isolation and culture of mesenteric arterial smooth muscle cells.

Male (6-wk or 12-wk-old) spontaneously hypertensive (SHR) or normotensive Wistar-Kyoto (WKY) rats were killed by cervical dislocation, a method approved under the United Kingdom Animals (Scientific Procedures) Act 1986. The investigation also conforms to the *Guide for the Care and Use of Laboratory Animals*, published by the National Institutes of Health (NIH Publication No. 85-23, revised 1996). Smooth muscle cells were isolated from small branches of mesenteric artery by enzymatic dissociation as previously described (13). Smooth muscle cells were separated by gentle trituration in medium 231 (Cascade Biologics, Nottingham, UK), supplemented with smooth muscle growth supplement, 100 IU penicillin, 100 μg/ml streptomycin, and 2.5 μg/ml amphotericin B. Cells plated onto coverslips were maintained in 5% CO_2_ in humidified air at 37°C.

#### Detection of arrestin protein expression.

Whole mesenteric trees were isolated from SHR and WKY rats, trimmed of all fat, and then homogenized in lysis buffer (50 mM Tris·HCl, pH 7.5, 150 mM NaCl, 1 mM EDTA, 0.25% wt/vol sodium dodecyl sulphate, 1% vol/vol IGEPAL CA-630). Insoluble material was removed via centrifugation, and the supernatants (40 μg of protein/lane) were separated using standard SDS-PAGE (10% gels). Following transfer to nitrocellulose membranes, arrestin expression was determined using an antibody (A1CT) that detects both arrestin 2 and to a lesser extent arrestin3, as described previously (1, 16, 21, 22, 33). Protein expression was quantified using the GeneGnome image analysis system (Syngene, Cambridge, UK).

#### Myography.

Contractile force recordings were made from 2- to 4-mm ring segments of third-order mesenteric arteries (diameter 150–250 μm at 6 wk, 200–300 μm at 12 wk) mounted in a Mulvany-Halpern wire myograph (Danish Myo Technology, Denmark) in bath solution (in mM: 134 NaCl, 6 KCl, 1 MgCl_2_, 1.8 CaCl_2_, 4 glucose, 6 mannitol, 10 HEPES, pH 7.4). For 60 mM K^+^ treatment, NaCl was reduced to 81 mM. All bath solutions contained l-*N*^ω^-nitroarginine methyl ester (l-NAME; 20 μM) to eliminate any effects of residual endogenous nitric oxide production. Solutions and drugs were added directly to the organ bath, maintained at 37°C.

#### Single-cell confocal imaging.

To examine the desensitization of ET_A_ and P2Y-receptor-stimulated PLC signaling, mesenteric smooth muscle cells (MSMC) were transfected with the pleckstrin-homology domain of PLCδ1 tagged with enhanced green fluorescent protein (eGFP-PH, 0.5 μg; an extensively characterized IP_3_ biosensor) (21–23, 33), using confocal imaging exactly as previously described. Receptor desensitization was determined using previously characterized protocols (21, 22). Changes in cytosolic eGFP-PH fluorescence are displayed as the fluorescence emission (F)/initial basal fluorescence (F_0_) (F/F_0_).

To assess the roles that arrestin proteins play in the regulation of ET_A_ and P2Y receptor PLC activity, MSMC were cotransfected with eGFP-PH and anti-arrestin2 (5′-GCCACUGACUCGGCUACAAtt-3′), anti-arrestin3 (5′-GCCUUCUGUGCCAAAUCUAtt-3′), or negative-control siRNA (all at 10 nM; Applied Biosystems, Warrington, UK). After 48 h, MSMC were loaded with Fura-Red (4 μM; 60 min) to enable simultaneous IP_3_ and Ca^2+^ measurements, and receptor desensitization was determined using a standard protocol (21, 22).

#### Data and statistical analysis.

Data presented are from vessels/cells obtained from at least three separate animals/preparations, expressed as means ± SE. Data were analyzed using one-way ANOVA as indicated, with appropriate post hoc testing (Prism, GraphPad, San Diego, CA).

## RESULTS

**Fig. 1.**
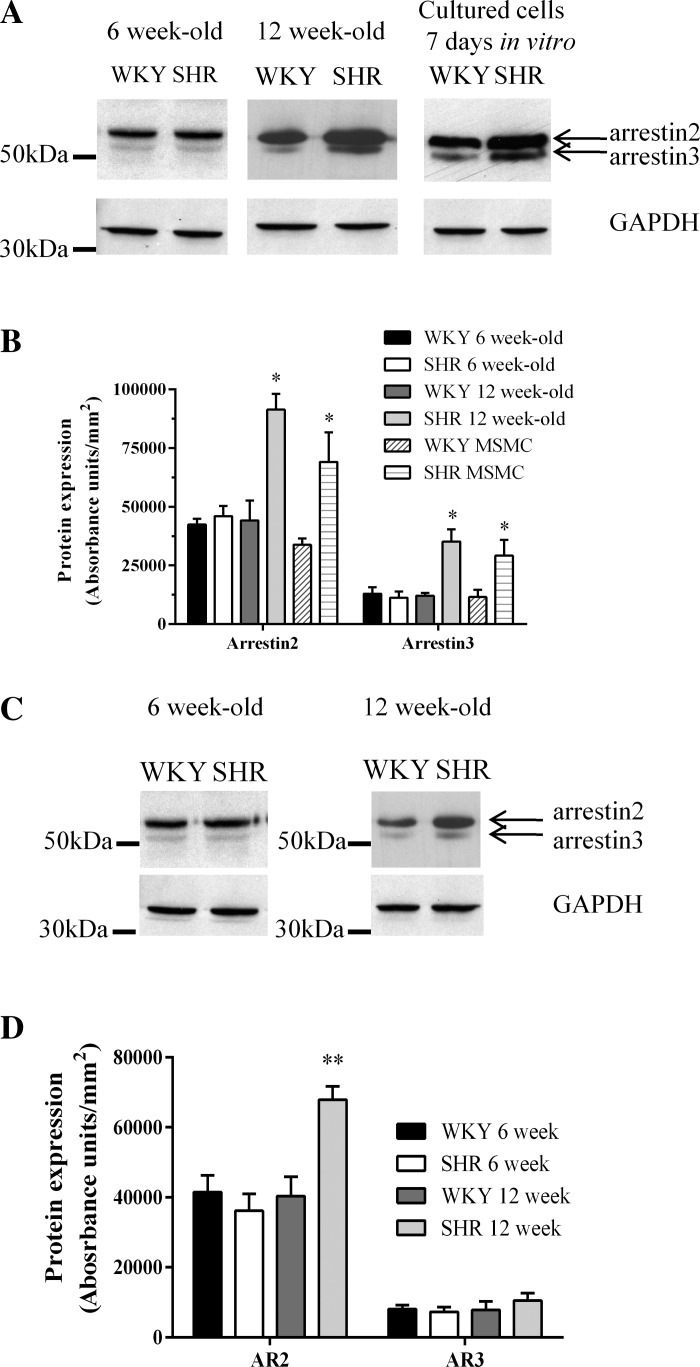
Increased arrestin expression in mesenteric and aortic arteries of 12-wk-old spontaneously hypertensive rats (SHR). Lysates (40 μg of protein) from the mesentery or aortae of SHR and Wistar-Kyoto (WKY) rats were subjected to SDS-PAGE separation and immunoblotting. *A*: representative immunoblots of mesenteric arrestin2, arrestin3, and GAPDH expression are shown from animals aged 6 or 12 wk or from cell lysates generated from SHR and WKY mesenteric smooth muscle cells (MSMC) after 7 days in culture. *B*: cumulative densitometric data showing means ± SE protein expression from 6-wk-old (*n* = 5) and 12-wk-old (*n* = 5) animals and 3 independent cell cultures. Statistical significance is indicated as *P < 0.05 SHR vs. WKY (unpaired *t*-test). *C*: representative immunoblots of aortic arrestin2 and arrestin3 GAPDH expression are shown from animals aged 6 or 12 wk. *D*: cumulative densitometric data showing means ± SE protein expression from 6-wk-old (*n* = 4) and 12-wk-old (*n* = 3) animals. AR, arrestin. Statistical significance is indicated as **P < 0.01 SHR vs. WKY (unpaired *t*-test).

**Fig. 2.**
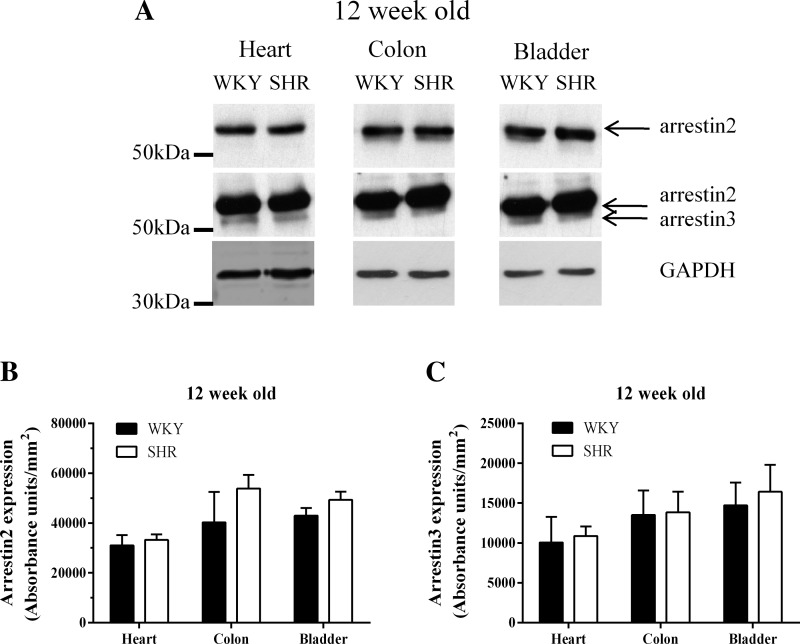
Profile of arrestin2 and arrestin3 expression in WKY and SHR heart, bladder, and colon. Tissues were dissected free of fat and homogenized, and 40 μg of each sample were loaded onto 10% SDS-PAGE gels for separation and immunoblotting as described in materials and methods. *A*: representative immunoblots of arrestin2, arrestin3, and GAPDH expression in the heart, colon, and bladder of 12-wk-old WKY and SHR animals. *B* and *C*: cumulative densitometric data showing means ± SE protein expression of arrestin2 (*B*) and arrestin3 (*C*), respectively.

Analysis of protein expression in the resistance arteries of the mesenteric tree of 6-wk-old animals showed there were no significant differences in arrestin expression in the vessels of SHR and WKY at this age (Fig. 1, *A* and *B*). Arrestin expression was also similar in aortae from 6-wk-old SHR and WKY animals (Fig. 1, *C* and *D*). At 12 wk of age, SHRs displayed significantly increased heart weight-to-body weight ratio compared with WKY rats (data not shown). In contrast to our findings in 6-wk-old animals, arrestin2 expression was significantly increased in both mesenteric vessels and aortae of 12-wk-old SHR compared with WKY rats (Fig. 1, *A*–*D*), with the highest expression being observed in SHR mesentery. Interestingly, arrestin3 expression was only significantly elevated in SHR mesentery (Fig. 1). No changes in arrestin2/3 expression were observed in other smooth muscle types (colon, bladder) or in the heart (Fig. 2), suggesting that the increases in arrestin isoform expression in arterial smooth muscle are tissue- and phenotype-specific changes. Importantly, the observed ex vivo differences in arrestin expression were preserved in mesenteric arterial smooth muscle cells (MSMC) isolated from 12-wk-old animals and maintained for 7 days under our culture conditions (Fig. 1, *A* and *B*).

**Fig. 3.**
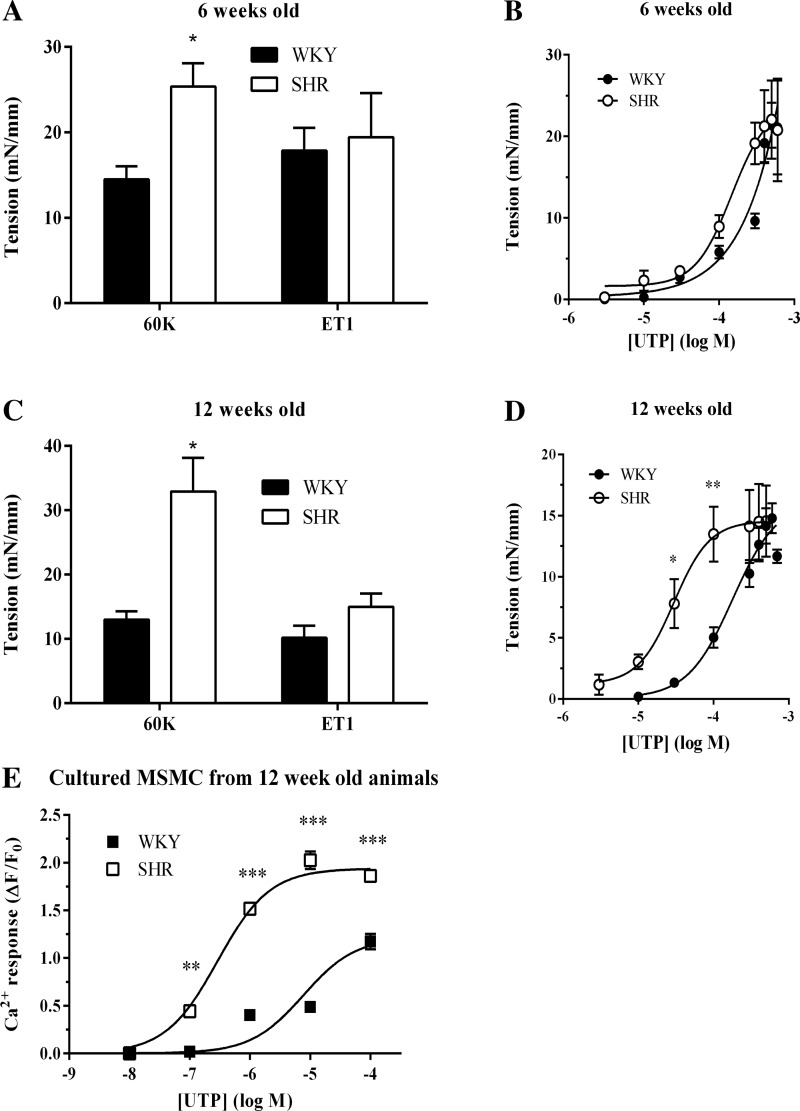
Comparison of contractile responses in mesenteric arteries from pre- and posthypertensive SHR and normotensive WKY rats. *A*: cumulative data showing third-order mesenteric arterial ring contraction (means ± SE) to K^+^ (60 mM) and endothelin-1 (ET1; 3 nM) for *n* ≥ 5 arterial preparations from at least 5 separate 6-wk-old SHR and WKY animals for each treatment. Statistical significance is indicated as *P < 0.05 SHR vs. WKY (unpaired *t*-test). *B*: concentration-dependency of UTP-stimulated contractions in SHR and WKY arteries from 6-wk-old animals (data are means ± SE for *n* ≥ 5 arterial preparations from at least 8 separate animals for each treatment). *C*: cumulative data showing the relative contractions of mesenteric arteries from 6-wk-old SHR and WKY animals (data are means ± SE for *n* = 7–19 arterial preparations from at least 7 separate animals for each treatment). Statistical significance is indicated as *P < 0.05 SHR vs. WKY (unpaired *t*-test). *D*: concentration-dependency of UTP-stimulated contractions in SHR and WKY arteries from 12-wk-old animals (data are means ± SE for *n* = 8 arterial preparations from at least 8 separate animals for each treatment). Statistical significance is indicated as *P < 0.05; **P < 0.01, SHR vs. WKY (two-way ANOVA and Bonferroni's post hoc test). *E*: concentration-dependency of UTP-stimulated Ca^2+^ signaling in MSMC prepared from 12-wk-old SHR and WKY animals. Data are means ± SE for *n* = 98–275 cells from at least 5 separate preparations for each animal strain. Statistical significance is indicated as **P < 0.01; ***P < 0.001, SHR vs. WKY (one-way ANOVA and Dunnett's post hoc test).

Since expression of arrestin2 and arrestin3 was elevated in small mesenteric arteries of 12-wk-old SHR and considering their key roles in suppressing the signaling of several contractile GPCRs within the vasculature (4, 14, 21, 22), we next compared the abilities of vasoactive agents to induce vasoconstriction in both SHR and WKY arteries. When normalized to vessel tension, contractions induced by K^+^ (60 mM) were similar in WKY to our previous findings in arterial preparations of adult male Wistar rats (21). However, K^+^-induced contractions were greater in SHR- compared with WKY-derived mesenteric vessels irrespective of age (Fig. 3, *A* and *C*). In both WKY- and SHR-derived vessels, contractions induced by K^+^ were completely ablated by preaddition of the L-type voltage-operated Ca^2+^-channel antagonist, nifedipine (data not shown).

No differences were observed when contractions were induced by ET1 or UTP (Fig. 3, *A* and *B*) in vessels from 6-wk-old animals. Similarly, at 12 wk of age, no differences in ET1 contractions were observed (Fig. 3*C*). Although maximal concentrations of UTP produced similar levels of vessel contraction, UTP-stimulated contractions more potently in vessels from 12-wk-old SHR compared with WKY rats with EC_50_ values of 29 and 179 μM, respectively (Fig. 3*D*). To determine whether the observed changes in the potency of vasoconstrictors to stimulate vessel contraction was reflected by changes in Ca^2+^ signaling, isolated MSMC from 12-wk-old animals were loaded with Fluo-4 and challenged with various agonists. UTP addition produced concentration-dependent increases in intracellular Ca^2+^ concentration ([Ca^2+^]_i_) that displayed greater potency in SHR cells than WKY (Fig. 3*E*), reflecting the changes seen for intact vessel contractile responses (Fig. 3*D*).

**Fig. 4.**
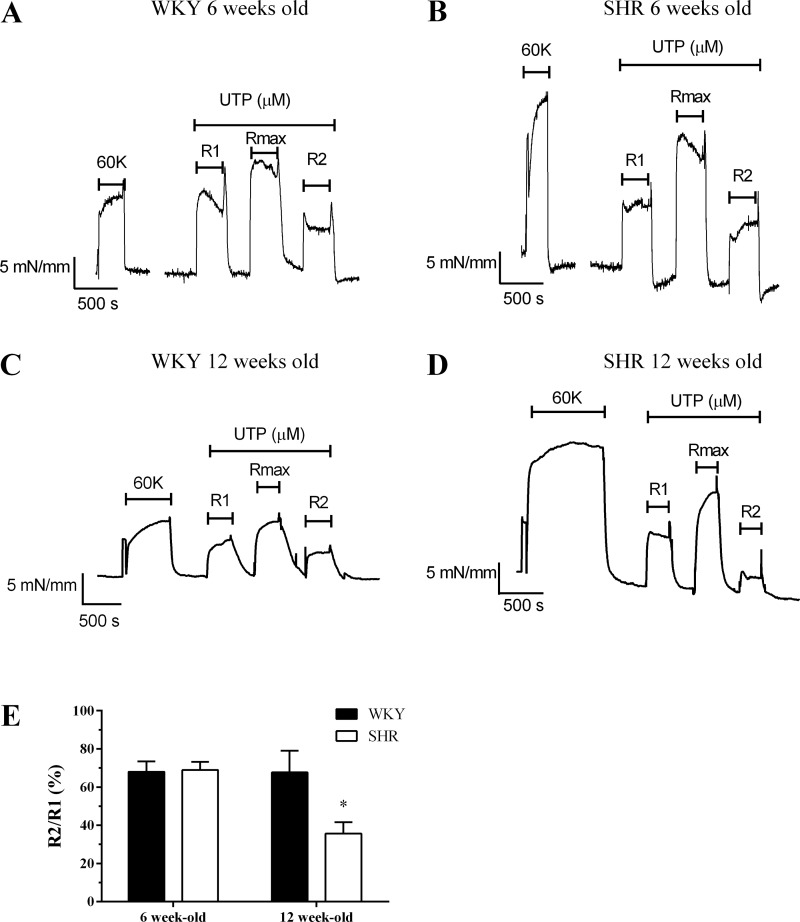
Profiling desensitization of UTP-mediated contractile responses in mesenteric arteries from pre- and posthypertensive SHR and normotensive WKY rats. Mesenteric arteries were subjected to the following desensitization protocol: 100 or 200 μM UTP (R1, for 5 min) challenge, followed by 5 min washout, prior to maximal UTP (300 or 500 μM, R_max_, 5 min) challenge, followed by a wash period of 5 min before further 100 or 200 μM UTP (R2, 5 min) exposure, for SHR or WKY vessels, respectively. Representative myograph traces are shown for arteries isolated from 6-wk-old WKY (*A*) and SHR (*B*) animals, or 12-wk-old WKY (*C*) or SHR (*D*) animals contracted with UTP. Desensitization of vessel contraction was determined as the relative change in R2 response compared with R1. Cumulative data (*E*) are expressed as means ± SE for the % change in R2 relative to R1; *n* = 7–9 vessels from ≥6 separate animals. Statistical significance is indicated as *P < 0.05 vs. WKY (unpaired *t*-test).

The ability of repeated UTP additions to diminish subsequent vessel contractions was compared in SHR- versus WKY-derived mesenteric arteries as an index of intact vessel “desensitization.” Artery rings were challenged with an approximate EC_50_ concentration of UTP, for 5 min before (R1) and after (R2) addition of a maximal UTP concentration for 5 min (R_max_). Arteries were washed for 5 min between R1 and R_max_, and R_max_ and R2 agonist challenges. The R2/R1 ratio is interpreted as an index of receptor desensitization (21, 22). Since the relative potency of UTP was different between rat strains, we applied an EC_50_ (R1 and R2) concentration of either 100 μM or 200 μM, and R_max_ concentration of 300 μM or 500 μM UTP for SHR and WKY vessels, respectively. In all cases, R2 responses were reduced compared with R1 after stimulation with a maximal agonist concentration (Fig. 4, *A*–*D*) and the reductions in R2/R1 ratio seen with WKY vessels (at both ages) were similar to that previously observed in vessels obtained from male adult Wistar animals (21). R2/R1 ratios were similar in vessels from 6-wk-old SHR and WKY rats (Fig. 4, *A*, *B*, and *E*); however, in vessels from 12-wk-old SHR the R2 response was further suppressed compared with age-matched WKY (decrease in R2 relative to R1: 64.3 ± 5.9% for SHR compared with 32.3 ± 11.3% for WKY; *P* < 0.05), indicating a significantly greater desensitization of UTP-stimulated SHR vessel contractions (Fig. 4*E*).

**Fig. 5.**
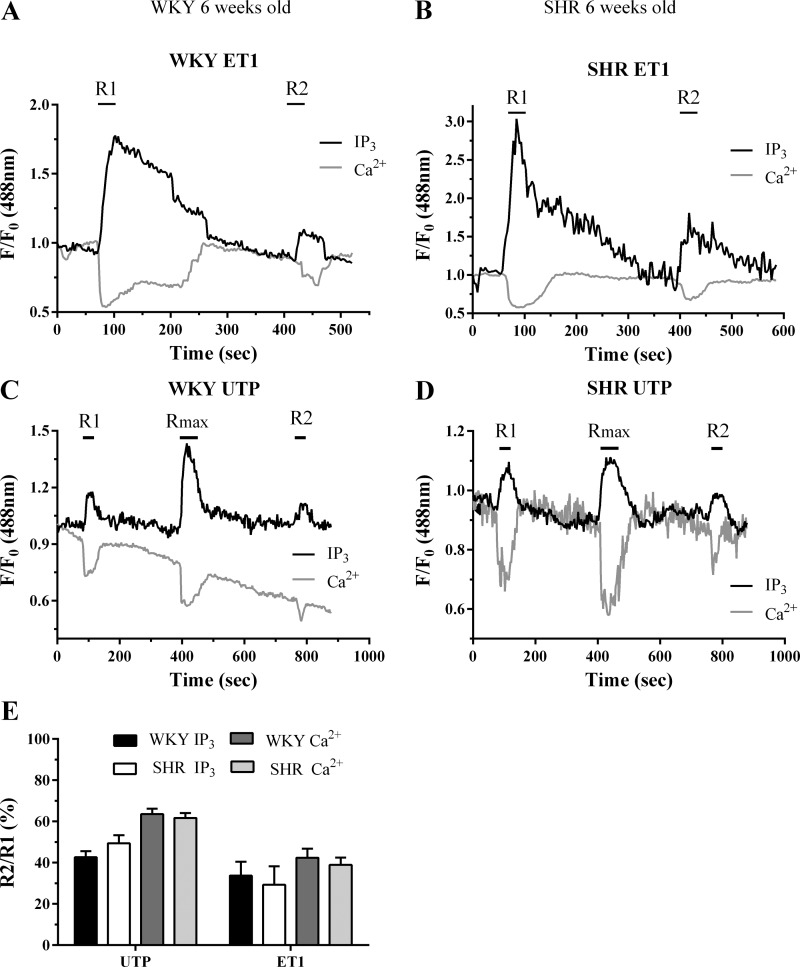
Assessment of ET1 and UTP-stimulated phospholipase C (PLC) signaling desensitization in SHR and WKY MSMC from 6-wk-old animals. MSMC were transfected with pleckstrin homology domain of PLCδ1 tagged with enhanced green fluorescent protein (eGFP-PH; 0.5 μg) before being subjected to the following desensitization protocols: For ET1, MSMC were stimulated with ET1 (50 nM, 30 s; R1); with 5 min washout before a second challenge (50 nM, 30 s; R2), while for UTP, MSMC were challenged with an ∼EC_50_ UTP concentration (10 μM) for 30 s before (R1) and after (R2) addition of a maximal UTP concentration (R_max_: 100 μM, for 1 min). Representative traces are shown from single cells isolated from WKY (*A* and *C*) and SHR (*B* and *D*) treated with either ET1 (*A* and *B*) or UTP (*C* and *D*). Receptor desensitization was determined as the relative change in R2 response compared with R1. Cumulative data (*E*) are expressed as means ± SE for the % change in R2 relative to R1; *n* = 7–20 cells from ≥6 separate animals. IP_3_, inositol 1,4,5-trisphosphate.

The extent of ET1- and UTP-induced PLC signaling desensitization was examined in MSMC transfected with eGFP-PH (0.5 μg) and loaded with the Ca^2+^-sensitive dye Fura-Red (4 μM, 60 min) to allow simultaneous measurement of changes in cellular IP_3_ and [Ca^2+^]_i_, using standard desensitization protocols (21, 22). For ET1, MSMC were challenged with a short desensitizing pulse of ET1 (50 nM, for 30 s, R1) followed by a wash period (5 min) and then a second ET1 challenge (50 nM, 30 s, R2). For cells prepared from 6-wk-old animals, initial ET1 challenge induced rapid, but transient translocations of eGFP-PH from the plasma membrane to the cytoplasm. Changes in eGFP-PH fluorescence were paralleled by changes in Fura-Red fluorescence, and responses returned to baseline within 5 min of agonist washout (Fig. 5, *A* and *B*). Changes in eGFP-PH and Fura-Red signals were reduced during a second (R2) ET1 challenge, indicative of diminished IP_3_ and Ca^2+^ increases in response to agonist re-challenge. For UTP, MSMC were challenged with a submaximal (approximate EC_50_) concentration of UTP (10 μM), for 30 s before (R1) and after (R2) addition of a maximal UTP concentration (100 μM, for 1 min, R_max_; to induce receptor desensitization). R2 values were attenuated compared with R1, indicating a desensitization of UTP-stimulated PLC signaling (Fig. 5, *C* and *D*).

**Fig. 6.**
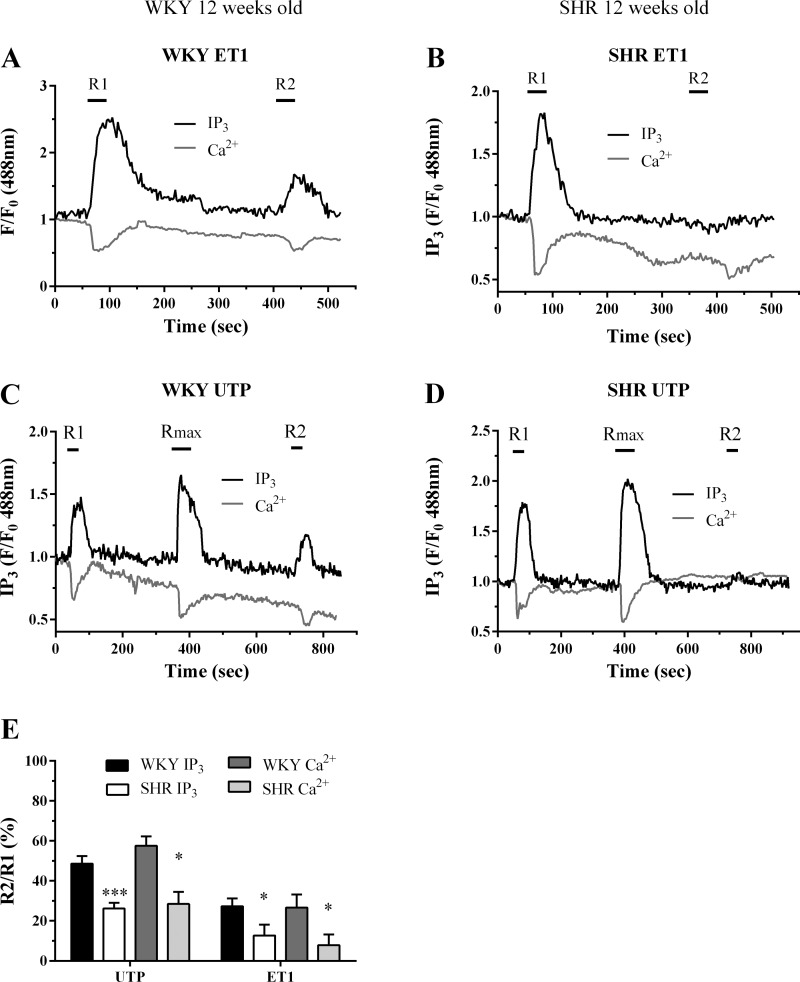
Assessment of ET1 and UTP-stimulated PLC signaling desensitization in SHR and WKY MSMC from 12-wk-old animals. MSMC were transfected with eGFP-PH (0.5 μg) before being subjected to the following desensitization protocols: For ET1, MSMC were stimulated with ET1 (50 nM, 30 s; R1); with 5 min washout before a second challenge (50 nM, 30 s; R2), while for UTP, MSMC were challenged with a ∼EC_50_ UTP concentration (10 μM) for 30 s before (R1) and after (R2) addition of a maximal UTP concentration (R_max_: 100 μM, for 1 min). Representative traces are shown from single cells isolated from WKY (*A* and *C*) and SHR (*B* and *D*) treated with either ET1 (*A* and *B*) or UTP (*C* and *D*). Receptor desensitization was determined as the relative change in R2 response compared with R1. Cumulative data (*E*) are expressed as means ± SE for the % change in R2 relative to R1; *n* = 10–18 cells from ≥4 separate animals. Statistical significance is indicated as *P < 0.05; ***P < 0.001 vs. WKY (one-way ANOVA and Dunnett's post hoc test).

The R2/R1 ratios obtained for ET1- and UTP-mediated desensitization in WKY MSMC (both at 6 and 12 wk; Figs. 5 and 6) were similar and were also comparable to those previously observed in adult Wistar MSMC (21, 22). Comparison of experiments undertaken in MSMC isolated from 6-wk-old animals showed that the extent of ET1- and UTP-induced receptor desensitization was comparable in SHR- and WKY-derived cells (Fig. 5*E*). When similar experiments were conducted in MSMC preparations from 12-wk-old animals, R2/R1 ratios were markedly decreased in SHR- compared with WKY-derived cells, indicating that a greater desensitization of ET1- and UTP-PLC signaling was observed in cells derived from 12-wk, but not 6-wk-old SHR (Fig. 6*E*).

As arrestin2 is a key regulator of P2Y receptor-stimulated PLC signaling in MSMC (21), we next examined whether the increased expression of arrestin2 in SHR vasculature is responsible for the increased extent of P2Y receptor desensitization observed in intact vessels and MSMC derived from 12-wk-old SHR. Therefore, to determine the effects of arrestin2 depletion on the desensitization of UTP-stimulated PLC signaling, MSMC were cotransfected with eGFP-PH (0.5 μg) and anti-arrestin2 or anti-arrestin3 siRNAs (10 nM), and loaded with Fura-Red, before being studied using the standard R1/R_max_/R2 desensitization protocol.

**Fig. 7.**
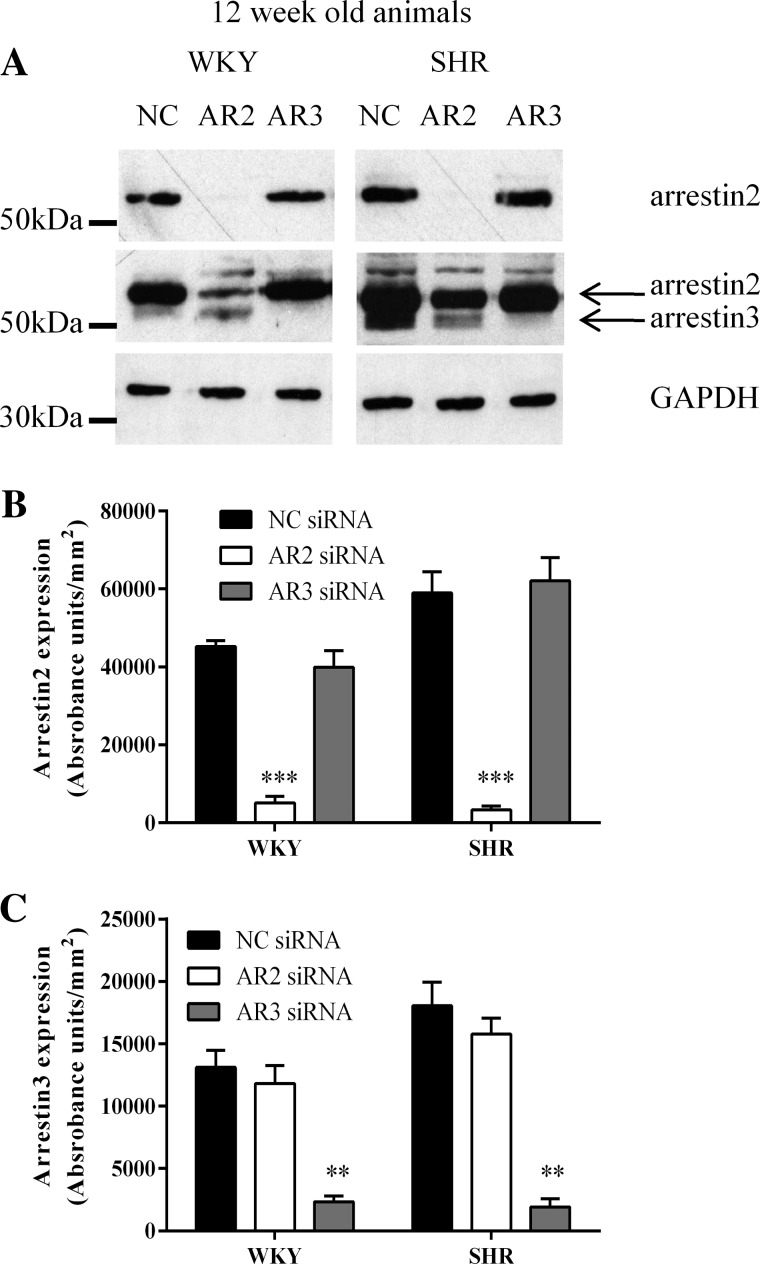
Specificity of arrestin isoformic knockdown. Arterial smooth muscle cells were transfected with 10 nM negative control (NC), anti-arrestin2 or anti-arrestin3 short-interfering RNAs (siRNAs) using the nucleofection technique. After 48 h, cells were lysed and 40 μg of each sample were loaded onto 10% SDS-PAGE gels for separation and arrestin expression was determined using immunoblotting as described in materials and methods. *A*: representative immunoblots showing the effect of individual siRNA treatments on arrestin expression. *B* and *C*: cumulative data (means ± SE for *n* = 5 cell preparations from 5 separate animals from each strain) showing the degree of arrestin2 (*B*) and arrestin3 (*C*) knockdown following siRNA treatments in comparison to GAPDH expression. Statistical significance is indicated as ***P < 0.001 for arrestin2 knockdown and **P < 0.01 for arrestin3 knockdown compared with negative control transfected cells (two-way ANOVA and Tukey's post hoc test).

**Fig. 8.**
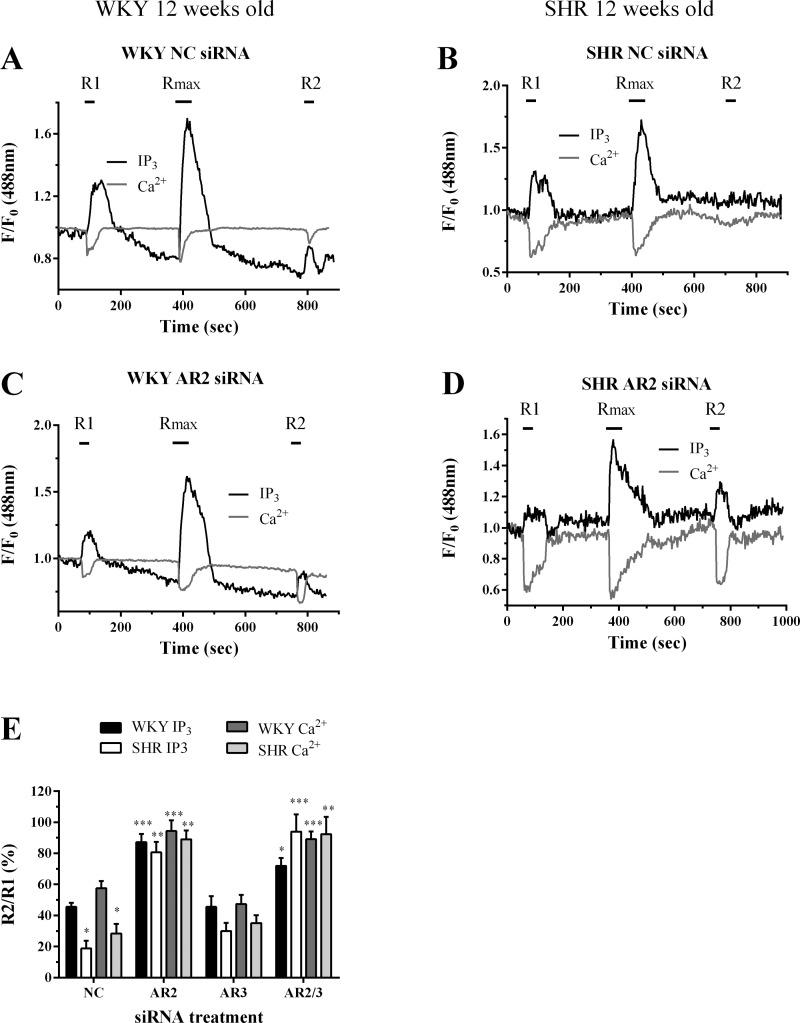
Knockdown of endogenous arrestin2 attenuates UTP-induced P2Y receptor desensitization. MSMC isolated from 12-wk-old animals were cotransfected with 0.5 μg eGFP-PH and 10 nM negative-control, anti-arrestin2, or anti-arrestin3 siRNAs. Cells were loaded with Fura-Red and subjected to the standard R1/R_max_/R2 desensitization protocol. Representative traces from single cells transfected with control (*A* and *B*), arrestin2 (*C* and *D*) are shown for WKY (*A* and *C*) and SHR (*B* and *D*) MSMC. Receptor desensitization was determined as the relative change in R2 response compared with R1. Cumulative data (*E*) are expressed as means ± SE for the % change in R2 relative to R1; *n* = 8–29 cells from ≥8 separate animals. Statistical significance is indicated as *P < 0.05; **P < 0.01, ***P < 0.001 vs. WKY negative-control treated cells (one-way ANOVA and Dunnett's post hoc test).

Western blotting analysis indicated that arrestin2 or arrestin3 siRNA treatments induced isoform-specific knockdowns of ≥85% and ≥80%, respectively, in both WKY- and SHR-derived cells (Fig. 7). Following MSMC transfection with negative-control siRNA, R2/R1 ratios in both WKY- and SHR-derived cells (Fig. 8, *A*, *B*, and *E*) were similar to those obtained in MSMC not transfected with siRNAs (see Fig. 6).

In agreement with our previous findings in cells from Wistar rats (21), transfection of WKY-derived MSMC with anti-arrestin2 siRNAs markedly reduced the decrease in R2 responses observed under control conditions, with only a 10–20% reduction in the IP_3_ response compared with R1, while for the UTP-stimulated Ca^2+^ signal the R2 response was little changed from R1 (Fig. 8, *C*, *D*, and *E*). A similar diminution of the UTP-stimulated receptor desensitization was also observed following knockdown of arrestin2 in SHR-derived MSMC, while knockdown of arrestin3 had no significant effect on P2Y-receptor desensitization in MSMC derived from WKY or SHR (Fig. 8*E*). Furthermore, combined suppression of arrestin2 and arrestin3 produced a reversal in P2Y receptor desensitization similar to that observed for arrestin2 knockdown alone (Fig. 8*E*). Collectively, these findings suggest that arrestin2 is the principal isoform regulating P2Y receptor desensitization in MSMC from both WKY and SHR.

**Fig. 9.**
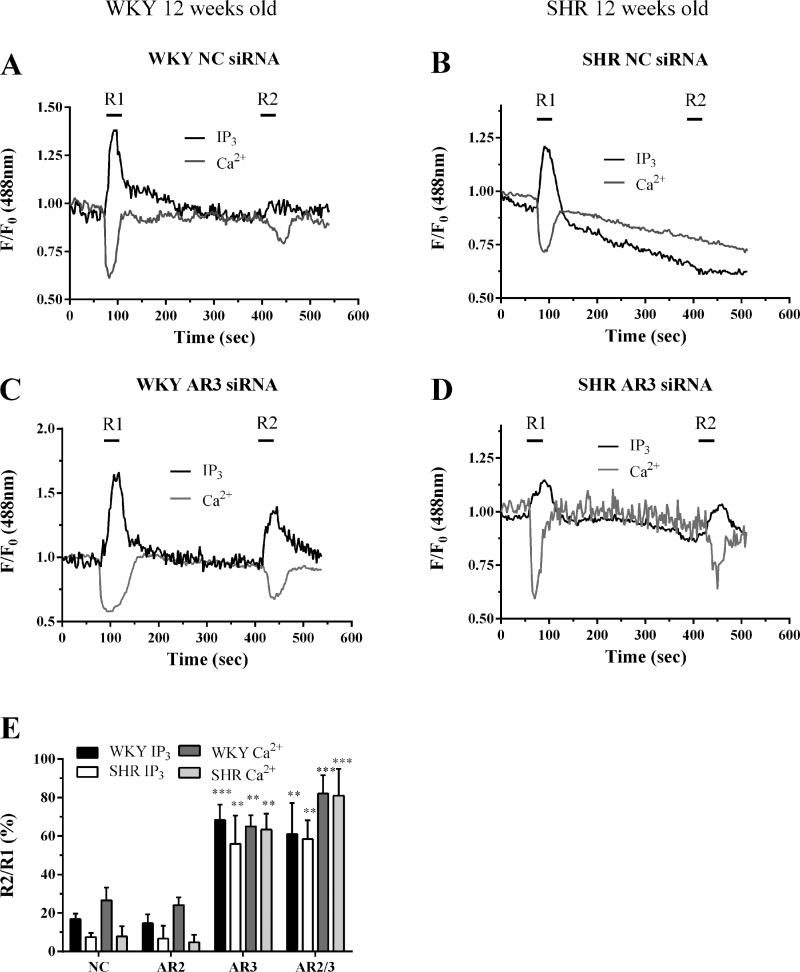
Knockdown of endogenous arrestin3 attenuates ET1-induced ET_A_ receptor desensitization. MSMC isolated from 12-wk-old animals were cotransfected with 0.5 μg eGFP-PH and 10 nM negative-control, anti-arrestin2, or anti-arrestin3 siRNAs. Cells were loaded with Fura-Red and subjected to the standard R1/R2 desensitization protocol. Representative traces from single cells transfected with control (*A* and *B*), arrestin2 (*C* and *D*) are shown for WKY (*A* and *C*) and SHR (*B* and *D*) MSMC. Receptor desensitization was determined as the relative change in R2 response compared with R1. Cumulative data (*E*) are expressed as means ± SE for the % change in R2 relative to R1; *n* = 5–17 cells from ≥8 separate animals. Statistical significance is indicated as **P < 0.01; ***P < 0.001 vs. WKY negative-control treated cells (one-way ANOVA and Dunnett's post hoc test).

Arrestin3 is known to regulate ET_A_ receptor desensitization in Wistar MSMC (22), therefore, we next examined whether the increased expression of arrestin3 in 12-wk-old SHR is responsible for the greater extent of ET_A_ receptor desensitization observed. To determine the effects of arrestins on ET1-stimulated PLC signaling, WKY- and SHR-derived MSMC were cotransfected with eGFP-PH (0.5 μg) and anti-arrestin2 or 3 siRNAs (as described above), prior to Fura-Red loading and being subjected to the R1/R2 desensitization protocol. Comparison of R2 and R1 responses from WKY-derived MSMCs transfected with either negative-control or arrestin2 siRNA indicated that ET1-stimulated PLC signaling was diminished by ∼80% following the second (R2) ET1 addition (Fig. 9*A*). These findings are similar to those observed in WKY- and Wistar-derived MSMC (22). In contrast, R2 responses were significantly greater after siRNA-mediated knockdown of arrestin3, indicating that ET_A_ receptor desensitization is diminished under these conditions (Fig. 9, *C*–*E*). Thus, the extent of ET1-induced receptor desensitization is attenuated following knockdown of arrestin3 in SHR-derived MSMC (Fig. 9*E*).

## DISCUSSION

Overactivity of vasoconstrictor GPCRs is one consequence of elevated circulating vasoconstrictor concentrations found during the development of hypertension (28, 29). The increased contractile force induced through increased activation of Gα_q_-coupled receptors, like ET_A_, P2Y_2_, and AT_1_, contributes significantly to the changes in peripheral arterial resistance associated with the disease (2). Since overstimulation of these signaling pathways is detrimental to health, it might be anticipated that some form of adaptive response is associated with disease, as an attempt to counteract the developing pathophysiology. Arrestin proteins play important roles in regulating GPCR signaling in vascular smooth muscle (4, 21, 22) and here we have examined whether their expression is altered during the development of hypertension. We report for the first time that arrestin2 and arrestin3 expression increases with the onset of hypertension and the greatest changes in expression occur in resistance arteries. Comparison of arrestin2/3 expression between mesenteric arterial and other smooth muscle cell types strongly suggests that the observed increase in protein expression is specific to arterial smooth muscle cells. Moreover, our data indicate that alterations in arterial arrestin2/3 expression appear before similar changes are detectable in the heart. Collectively, our observations indicate that, at least in the SHR model of hypertension, specific alterations in arrestin2/3 expression are observed in resistance arteries during the early stages of hypertension. These changes may represent early adaptive mechanisms to counteract enhanced vasoconstrictor signaling.

A number of previous studies have examined differences between SHR and WKY contractile responses to a variety of vasoconstrictors, often with contradictory results (6). Therefore, we have conducted experiments to determine the profile of contractile responses to a panel of vasoconstrictors in these rat strains. Most strikingly, SHR were more responsive to high (60 mM) K^+^ stimulation than WKY, irrespective of age. As K^+^-stimulated contractions were nifedipine sensitive, it appears likely that SHRs express more L-type voltage-gated Ca^2+^ channels in their arterial smooth muscle than WKY, both before and after onset of hypertension, in agreement with previous findings (26, 27). Considering the propensity to normalize vessel contraction data to this index, our findings have obvious implications, suggesting that in this animal model, myography data are most reliably normalized to vessel tension rather than K^+^-induced contraction. Aside from the alterations in sensitivity to a depolarizing stimulus, no changes in ET1-stimulated vessel contraction were observed at 6 or 12 wk. Conversely, the potency of UTP to induce vessel contraction was markedly shifted in 12-wk-old SHR, which was underpinned by a corresponding change in Ca^2+^ signaling within MSMC. A similar alteration in contractile and Ca^2+^ signaling has previously been reported following noradrenaline challenge in SHR-derived mesenteric arteries and was linked to an increased capacity to store Ca^2+^ in the sarcoplasmic reticulum (19). These findings suggest that increased Ca^2+^ store loading and subsequent release is likely to be responsible for the enhanced potency of UTP in MSMC isolated from 12-wk-old SHR.

Previously we have shown that by applying a variation of our standard R1/R_max_/R2 protocol we can quantify an index of desensitization associated with UTP-induced contractions in intact mesenteric vessel rings (21). Applying the same protocol here we have demonstrated age-dependent changes in UTP-induced vessel desensitization, with increased desensitization in 12-wk-old SHR animals compared with both age-matched WKY and younger animals of both strains. Although reduced vessel contractility may reflect a loss in responsiveness of a wide variety of contractile pathways, the PLC signaling pathway contributes significantly to this process. It is therefore interesting to note that the degree of desensitization of UTP-induced vessel contraction strongly correlates with the highest expression of a key protein known to regulate negatively UTP-induced PLC signaling in the mesentery, arrestin2 (20, 21). In support of this, we also showed that at 6 wk of age, when levels of arrestins are identical in SHR and WKYs, the degree of UTP-induced desensitization, as measured by simultaneous confocal imaging of IP_3_ and [Ca^2+^]_i_, was indistinguishable. Also, in agreement with our myography data, desensitization of the response to UTP was markedly enhanced in MSMC from 12-wk-old SHR. Moreover, the finding that siRNA-mediated knockdown of arrestin2 largely reversed the desensitization of UTP-stimulated P2Y receptor-PLC signaling, yet arrestin3 knockdown was ineffective, confirms the key roles of these proteins in the regulation of the vascular P2Y receptor in both WKY and SHR. Collectively, these data suggest that increased arrestin expression, which accompanies the development of hypertension in SHR, mediates an increase in the desensitization of both UTP-mediated PLC activation and vessel contractility.

As arrestin3 expression is enhanced in mesenteric vessels and MSMCs from 12-wk-old SHR, and both proteins are known to regulate ET_A_ receptor function (22), we have also examined whether developing hypertension has effects on ET1-induced PLC signaling. We find that in 6-wk-old SHR and WKY MSMC, when arrestin2/3 expression is unaltered, the levels of desensitization of the receptor-PLC signaling by ET1 are also identical, and similar to data previously obtained in adult Wistar MSMC (22). The ability of ET1 to induce a greater extent of desensitization of ET_A_ receptor-PLC signaling is only evident in vessels and MSMCs from older SHR animals. siRNA-targeted knockdown of arrestin3 substantially reversed the desensitization of ET1-stimulated PLC signaling in both SHR and WKY MSMC, while arrestin2 knockdown did not. That arrestin3 depletion was able only to partially reverse ET1-induced ET_A_ receptor desensitization indicates that other desensitization mechanisms also regulate ET_A_ receptor-PLC signaling. Taken together, these data further support a key role for arrestin3 in the regulation of ET_A_ receptor signaling in MSMC in both health and disease.

The development of hypertension is a multifactorial process initiated by the actions of many different stimuli, including sympathetic drive and endothelial dysfunction, leading to enhanced circulating levels of a number of vasoconstrictors (7). It is therefore unsurprising that the expression of key regulators of GPCR signaling, such as arrestin proteins is altered in cells/tissues primarily affected by changes in blood pressure, presumably in an attempt to combat enhanced vasoconstrictor signaling in arterial smooth muscle. Presently, it remains uncertain whether these adaptive changes in GPCR regulatory proteins are beneficial or detrimental to vascular pathophysiology. Certainly, it might be expected that elevated arrestin isoformic expression can counteract vasoconstrictor-induced hypertensive changes observed in SHR. However, the situation is complicated by the fact that changes in arrestin2/3 expression can also affect other signaling pathways, some of which play potentially important roles in regulating blood pressure. For example, arrestin proteins are known to negatively regulate β-adrenergic receptor signaling, and their increased expression may blunt the capacity to induce vasorelaxation (10).

Our finding that arrestin2/3 expression is enhanced in resistance arteries following the development of hypertension raises the question of what the potential consequences are for vascular pathophysiology. Considering the multifaceted involvement of arrestin proteins in the regulation of GPCR signaling it might be predicted that the activity of virtually all GPCRs within arterial smooth would be affected. However, since changes in vascular GRK expression seem restricted to GRK2, this suggests that GPCRs which primarily recruit GRK2, such as AT_1_, ET_A_, P2Y_2_, and α_1D_-adrenergic receptors (4, 21, 22, 24), would be most affected. Increased GPCR signaling likely corresponds with enhanced arrestin recruitment, which would augment arrestin-dependent signaling in arterial smooth muscle. Furthermore, arrestins are known to regulate vasoconstrictor-stimulated mitogen-activated protein kinase (MAPK) signaling in vascular smooth muscle (14, 20), which implies that their enhanced expression may underlie the elevated MAPK signaling reported in hypertensive SHR(11, 17, 32). Moreover, arrestins can regulate MAPK-dependent and -independent signaling pathways to promote cell motility (5, 18) and proliferation (30, 31), two integral processes for vascular remodeling. Therefore, it seems probable that increased arrestin expression observed early in the development of hypertension has potential to augment vasoconstrictor-induced vascular damage. Indeed, the previous finding that arrestin3 plays a key role in the smooth muscle cell growth and migration during atherosclerosis supports this notion (15).

In summary, arrestin protein expression increases in resistance arteries at an early stage in the development of hypertension, possibly representing an early adaptive response to limit vasoconstrictor-stimulated vessel contractions. However, since arrestin proteins can enhance vascular smooth muscle migration and proliferation, their increased expression may potentially exacerbate vasoconstrictor-mediated vascular remodeling and hypertension. Indeed, a greater understanding of the contribution of elevated GRK2/arrestin expression to such remodeling processes should clarify the future potential to target these proteins in the clinical management of hypertension.

## GRANTS

This work was supported by British Heart Foundation Grants PG/11/60/29007 and RG06/008/22062 (to J. M. Willets and R. A. J. Challiss).

## DISCLOSURES

No conflicts of interest, financial or otherwise, are declared by the author(s).

## AUTHOR CONTRIBUTIONS

J.M.W. and R.A.J.C. conception and design of research; J.M.W., C.A.N., R.D.R., and C.P.N. performed experiments; J.M.W., C.A.N., R.D.R., C.P.N., and R.A.J.C. analyzed data; J.M.W., C.A.N., R.D.R., C.P.N., and R.A.J.C. interpreted results of experiments; J.M.W., C.A.N., and R.D.R. prepared figures; J.M.W. and R.A.J.C. drafted manuscript; J.M.W., C.A.N., R.D.R., C.P.N., and R.A.J.C. edited and revised manuscript; J.M.W., C.A.N., R.D.R., C.P.N., and R.A.J.C. approved final version of manuscript.

## Glossary

ATII: Angiotensin II
ET1: Endothelin-1
eGFP-PH: Pleckstrin homology domain of phospholipase Cδ1 tagged with enhanced green fluorescent protein
GPCR: G protein-coupled receptor
GRK: G protein-coupled receptor kinase
IP_3_: Inositol 1,4,5-trisphosphate
MAPK: Mitogen-activated protein kinase
MSMC: Mesenteric smooth muscle cells
PLC: Phospholipase C
SHR: Spontaneously hypertensive rat
siRNA: Short-interfering RNA
UTP: Uridine 5′-triphosphate
WKY: Wistar-Kyoto rat
